# Sequential bilateral rTMS combined with fluoxetine for anhedonic depression in adolescents: clinical efficacy and peripheral inflammatory profiles

**DOI:** 10.3389/fpsyt.2026.1925491

**Published:** 2026-08-19

**Authors:** Ou Zhang, Chao Zhou, Chen Zhang, Wang Qi, Lin Fan, Xu Han

**Affiliations:** 1 Department of Psychiatry and Psychology, General Hospital of Northern Theater Command, Shenyang, Liaoning, China; 2 Xingcheng Special Service Sanatorium, Joint Logistics Support Force, Xingcheng, Liaoning, China; 3 Department of General Practice, General Hospital of Northern Theater Command, Shenyang, Liaoning, China

**Keywords:** adolescent, anhedonic, depression, fluoxetine, inflammation, repetitive transcranial magnetic stimulation

## Abstract

**Objective:**

Adolescent anhedonic depression often fails to respond fully to fluoxetine monotherapy, driven by chronic peripheral inflammation and dysfunctional reward circuits. This study aimed to compare clinical efficacy and inflammatory signatures of fluoxetine alone versus sequential bilateral rTMS plus fluoxetine in adolescents with anhedonic major depression.

**Methods:**

This single-centre 4-week randomised controlled trial recruited 86 Han Chinese adolescents aged 13–18 years meeting DSM-5 major depression with prominent anhedonia, allocated 1:1 to combination (active bilateral rTMS + fluoxetine) and control (sham rTMS + fluoxetine) groups. Eighty-one participants completed treatment. The primary endpoint was the change in ASA-C score; secondary endpoints included changes in HAMD-24 total score, WCST cognitive indices, and plasma IL-1β, IL-6, and TNF-α levels. ANCOVA adjusted for baseline values for intergroup comparisons, with Pearson correlation analysing symptom-cytokine relationships. Adverse events were prospectively monitored.

**Results:**

Both groups presented significant within-group improvements in depressive/anhedonic symptoms, cognition and proinflammatory cytokines post-treatment. After baseline adjustment, the combination group showed superior reductions in ASA-C, HAMD-24 and all three cytokines, as well as better executive function (all *P* < 0.001). Symptom relief magnitudes moderately correlated with cytokine declines in the combination arm. All adverse events were mild and transient; overall event rates did not differ statistically between groups (*P* = 0.079).

**Conclusion:**

Adjunct sequential bilateral rTMS yields synergistic benefits for adolescent anhedonic depression by ameliorating core symptoms, restoring executive function and attenuating systemic inflammation. Peripheral IL-1β, IL-6 and TNF-α are viable biomarkers for therapeutic monitoring, and this combined regimen is safe for adolescents with residual anhedonia after fluoxetine monotherapy.

## 1 Introduction

Adolescent depression represents a severe and growing public health crisis worldwide, distinguished from adult depressive episodes by prominent developmental vulnerability, heterogeneous symptom presentations, and poor long-term functional outcomes if left inadequately treated (1). Previous reports have found that approximately one-fifth of children and adolescents worldwide suffer from depression or depressive symptoms, and this proportion continues to rise year by year (2). In contrast to classic depressive phenotypes featuring predominant low mood, anhedonic depression represents a clinically distinct subtype marked by severe loss of interest and pleasure in formerly rewarding activities, a core symptom that frequently persists despite partial alleviation of affective distress following standard antidepressant monotherapy (3, 4). For adolescents, persistent anhedonia interferes with peer interaction, academic engagement and identity formation, substantially elevating risks of self-harm, school dropout and recurrent mood disorders into young adulthood—even after partial symptom relief with first-line fluoxetine monotherapy (5–7).

Despite its clinical significance, anhedonia remains notoriously resistant to first-line selective serotonin reuptake inhibitors (SSRIs), leaving a substantial proportion of young patients with residual symptoms and unmet therapeutic needs (6, 8). Fluoxetine is a SSRI approved by the U.S. Food and Drug Administration (FDA) for the pharmacological treatment of major depressive disorder (MDD) in adolescents (9). A large body of robust research evidence demonstrates that this drug achieves significantly greater improvements in overall depressive symptoms compared with placebo (10). Animal studies have shown that fluoxetine alleviates anhedonia via anti-inflammatory effects and enhanced neuroplasticity (11). Nevertheless, multiple trials have documented limited efficacy of fluoxetine alone in mitigating anhedonic symptoms (12, 13). A key contributing factor to this treatment gap lies in the multifactorial pathophysiology of adolescent anhedonic depression, which extends far beyond central serotonin dysregulation. Accumulating translational and clinical evidence identifies chronic low-grade systemic inflammation as a core mechanistic mediator. Specifically, elevated peripheral proinflammatory cytokines disrupt the mesolimbic reward circuit, compromise dopaminergic neurotransmission, and attenuate hedonic processing—thereby establishing a biological phenotype that exhibits limited responsiveness to monoamine-modulating pharmacotherapies (12, 14, 15). Conventional antidepressants possess limited anti-inflammatory capacity (16, 17), which may account for their inadequate efficacy in ameliorating reward dysfunction underlying inflammation-linked anhedonia. This underscores an unmet clinical need for synergistic combination therapies capable of concurrently regulating inflammatory signaling cascades and reward-associated cerebral networks.

Repetitive transcranial magnetic stimulation (rTMS) has emerged as a well-tolerated, non-invasive neuromodulation technique for depression across age groups (18). Previous studies have demonstrated that rTMS can alleviate inflammatory activation of microglia in the brain and reduce the levels of peripheral inflammatory cytokines in patients with depression (19). Conventional unilateral rTMS targeting the left dorsolateral prefrontal cortex (DLPFC) yields improvements in general depressive severity, yet its effects on anhedonia remain inconsistent, particularly among adolescents with inflammatory activation (20). Sequential bilateral rTMS, which sequentially modulates hypoactive left DLPFC and overactive right DLPFC, is hypothesised to better rebalance prefrontal-limbic reward circuits relative to unilateral protocols (21).

We hypothesize that sequential bilateral rTMS confers synergistic therapeutic effects when combined with fluoxetine. Specifically, fluoxetine modulates central serotonergic homeostasis, whereas bilateral neuromodulation restores reward circuit activity and mitigates pro-inflammatory stress. Together, the two interventions concurrently target the dual neurochemical and inflammatory pathological mechanisms underlying anhedonic depression in adolescents. To date, few randomised controlled trials (RCTs) have specifically focused on adolescent anhedonic depression, and no prospective controlled study has systematically evaluated the combined regimen of sequential bilateral rTMS plus fluoxetine in this patient population.

This randomised controlled trial aimed to compare the clinical efficacy of fluoxetine monotherapy versus sequential bilateral rTMS adjunct to fluoxetine in adolescents diagnosed with anhedonic depression. We further quantified peripheral inflammatory cytokines at baseline and post-intervention to delineate distinct inflammatory signatures associated with treatment response. The present findings aim to yield actionable clinical evidence for optimized combination therapies targeting anhedonia among young individuals with depression.

## 2 Materials and methods

### 2.1 Study design

This single-center RCT was conducted in the Department of Psychiatry and Psychology, General Hospital of Northern Theater Command between June 2024 and December 2025. The trial strictly followed the Consolidated Standards of Reporting Trials (CONSORT) 2010 statement for RCT reporting. The present study was approved (Approval No. Y-2021-023) by the Medical Ethics Committees of the General Hospital of Northern Theater Command. This trial was prospectively registered with the Chinese Clinical Trial Registry (Registration Number: ChiCTR2400081227). Participants and their legal guardians provided written informed consent. The study adopted a 1:1 random allocation ratio with blinded outcome assessors. The trial timeline included a 4-week acute intervention phase; clinical evaluations and peripheral inflammatory biomarker testing were performed only at baseline and after 4 weeks of intervention.

### 2.2 Participants

#### 2.2.1 Inclusion criteria

① Age range 13–18 years and of Han Chinese ethnicity;

② Met diagnostic criteria for MDD according to the Diagnostic and Statistical Manual of Mental Disorders, Fifth Edition (DSM-5), confirmed via structured clinical interview for DSM-5 (SCID-5);

③ The Chinese Anhedonia Scale for Adolescents (ASA-C) total score ≥ 21 at baseline screening. Marked anhedonia was identified by a total score of more than 21 on the ASA-C (22);

④ Total score of Hamilton Depression Rating Scale for Adolescents (HAMD-24) ≥ 20 at baseline;

⑤ Participants with no prior antidepressant exposure within 3 months before screening; if previously medicated, a washout period of at least 5 half-lives was enforced; participants with a documented history of non-response to prior fluoxetine trials were excluded.

⑥ Ability to attend all scheduled intervention and follow-up visits, complete scale assessments and peripheral blood collection as required.

#### 2.2.2 Exclusion criteria

① Comorbid diagnosis of bipolar disorder, schizophrenia spectrum disorders, autism spectrum disorder, severe conduct disorder or substance use disorder;

② History of epilepsy, febrile convulsions, intracranial trauma, intracranial metal implants, pacemaker implantation, or any absolute contraindication to TMS;

③ Current use of immunosuppressants, glucocorticoids, nonsteroidal anti-inflammatory drugs taken for >7 consecutive days within 2 weeks before enrollment; chronic inflammatory autoimmune diseases (rheumatoid arthritis, inflammatory bowel disease, systemic lupus erythematosus etc.);

④ Severe somatic diseases including uncontrolled thyroid dysfunction, chronic hepatic/renal failure, hematological disorders, active infectious diseases within 4 weeks before screening;

⑤ Female adolescents with irregular menstruation and unprotected sexual activity;

⑥ Previous receipt of rTMS, electroconvulsive therapy (ECT) or transcranial direct current stimulation (tDCS) within 6 months prior to screening;

⑦ Suicidality rated as high risk (HAMD-24 suicide item score = 3) requiring urgent ECT or inpatient crisis intervention during screening.

### 2.3 Interventions

Two parallel treatment arms were established: experimental group (sequential bilateral rTMS + fluoxetine) and control group (fluoxetine monotherapy + sham rTMS). No additional antidepressants, anxiolytics or anti-inflammatory agents were permitted during the trial period.

Oral fluoxetine hydrochloride capsules were administered at a fixed daily dose of 20 mg. All participants received study medication from a single consistent batch throughout the entire trial, and the dosage remained constant for the duration of the experimental intervention.

The stimulation sites were the left and right dorsolateral prefrontal cortices (L-DLPFC and R-DLPFC). All rTMS treatments were delivered using a magnetic stimulator (YRD CCY-II, Wuhan Yiruide Medical Treatment Equipment New Technology Co., Ltd, Wuhan, China) with a circular coil. Motor threshold (MT) was determined at baseline for each participant by measuring resting motor threshold (RMT) of the abductor pollicis brevis muscle, defined as the minimum stimulus intensity evoking visible muscle twitch in ≥5 out of 10 consecutive pulses (23). The standard 5.5 cm localization method was adopted to identify the bilateral DLPFC stimulation sites (23). For the sequential bilateral rTMS protocol, each treatment session commenced with low-frequency (1 Hz) inhibitory stimulation targeting the R-DLPFC, followed immediately by high-frequency (10 Hz) excitatory stimulation over the L-DLPFC without an inter-session interval. The asymmetric dosing (900 pulses for R-DLPFC vs. 3,000 for L-DLPFC) follows established physiological and clinical rationales: ① Physiological basis:​ Low-frequency (1 Hz) rTMS induces inhibitory long-term depression (LTD)-like effects in the hyperactive R-DLPFC, while high-frequency (10 Hz) rTMS enhances excitatory long-term potentiation (LTP)-like plasticity in the hypoactive L-DLPFC (18). ② Clinical precedence:​ This pulse ratio aligns with validated sequential bilateral protocols targeting interhemispheric imbalance in depression (21, 31).

Individual stimulation intensity was calibrated to 100% RMT, which was measured over the primary motor cortex of each participant’s dominant hand before the first treatment. The R-DLPFC received a total of 900 pulses per session, each session consisted of 30 trains of 30 pulses each, with 10 s inter-train intervals. The L-DLPFC received 3000 pulses per session, each session consisted of 60 trains of 50 pulses each, with 30 s inter-train intervals. Both stimulations were administered once daily, 5 days per week (Monday to Friday) for 4 consecutive weeks.

In the control group, sham stimulation was administered. The coil was placed at a 90° angle relative to the scalp to prevent the signal from penetrating the skull. Other stimulation parameters, including frequency and intensity, were set identically to those in the observation group.

### 2.4 Outcome

Primary and secondary outcomes were assessed at baseline (pre-intervention) and week 4 of treatment.

#### 2.4.1 Primary outcome

Change in anhedonia severity measured by the ASA-C (22). This 14-item self-report scale adopts a 4-point Likert scale (0 = never to 3 = always), with three positive items reverse-scored; total scores range from 0 to 42, where higher scores reflect more severe anhedonia.

#### 2.4.2 Secondary outcomes

Change in depressive symptom severity measured by the HAMD-24 (24). The HAMD-24 is a widely used clinician-administered tool for assessing depressive symptom severity in adults. Most items are scored on a 5-point Likert scale (0–4), with a few using a 3-point scale (0–2). It assesses seven symptom domains: anxiety-somatization, cognitive disturbance, retardation, desperation, weight, sleep disturbance, and diurnal variation. Higher scores indicate more severe symptoms.

Cognitive function was evaluated using Wisconsin Card Sorting Test (WCST). WCST is a neuropsychological test assessing “set-shifting” which involves the cognitive functions of attention, working memory, and visual processing. The indices from the WCST included percentage of correct responses (PCR) and the percentage of conceptualization levels (PCL) (25).

Fasting cubital venous blood (5 mL) was collected using EDTA anticoagulant tubes, placed on ice immediately after collection, centrifuged within 30 min at 3000 r/min for 10 min at 4 °C to separate plasma, aliquoted into cryovials and stored at −80 °C ultra-low temperature freezer until batch testing. Concentrations of three core inflammatory cytokines were detected via double-antibody sandwich enzyme-linked immunosorbent assay (ELISA) kits purchased from Boster Biological Technology Co., Ltd (China): interleukin-1β (IL-1β), interleukin-6 (IL-6), and tumor necrosis factor-α (TNF-α). All testing steps strictly followed kit manufacturer operating instructions.

Adverse effects were systematically monitored and recorded throughout the intervention period.

### 2.5 Sample size calculation

Sample size calculation was conducted using PASS 15 statistical software based on the primary endpoint—the change in ASA-C anhedonia scale score at week 4—under a two independent samples t-test framework. The reference effect parameters were obtained from our preliminary trial data: the combination intervention group exhibited a mean ASA-C score reduction of 6.0 points (standard deviation [SD] = 3.2), while the antidepressant monotherapy group showed a mean reduction of 3.4 points (SD = 2.9). With a two-sided significance level α set at 0.05 and a statistical power of 1−β = 0.80, the minimal evaluable sample size without dropout correction was calculated to be 39 subjects per group. Accounting for an estimated overall 10% loss to follow-up and participant dropout over the 4-week trial period, the target recruitment number was inflated to 43 adolescents per group, with a total planned sample size of N = 86 adolescents in this study.

### 2.6 Randomization and blinding

Participants were randomly allocated at a 1:1 ratio into either the experimental group or the control group, with 43 participants in each group. Group assignment was performed via a computer-generated simple randomisation sequence. All outcome assessors remained unaware of the treatment allocations throughout the trial. Before study commencement, they received clear instructions not to disclose or discuss participants’ diagnostic information with the participants themselves or any personnel involved in group assignment. Given the inherent differences in stimulation protocols and parameters between the two interventions, blinding of the treating clinicians was not feasible in this study.

### 2.7 Statistical analysis

All statistical analyses were conducted with SPSS 20.0, and a two-sided α=0.05 was defined as the statistical significance threshold. Continuous data were summarized as mean ± SD, while categorical data were expressed as frequencies and percentages. Baseline demographic, clinical scale, cognitive and inflammatory biomarker characteristics between the two group were contrasted via independent samples t-test for normally distributed variables, and Chi-square test for categorical variables. Analysis of covariance (ANCOVA) was the primary analytical approach to compare intergroup differences in the week-4 post-intervention values of primary outcomes (ASA-C) and secondary outcomes (HAMD-24, WCST indices, IL-1β, IL-6, TNF-α), with the corresponding baseline value of each indicator incorporated as a covariate to eliminate baseline imbalance bias. The incidence of adverse events across two groups was compared using Chi-square test. The correlations between the change values of HAMD-24 scores, ASA-C scores and the change values of IL-1β, IL-6, TNF-α were examined via Pearson correlation analysis.Pearson correlation analyses between inflammatory cytokines and clinical scales were conducted as exploratory analyses and were not adjusted for multiple comparisons.

## 3 Results

### 3.1 Characteristics of participants

A total of 97 adolescent patients were initially screened for eligibility in this study. Of these, 11 individuals were excluded prior to randomization due to failure to meet the predefined inclusion criteria or voluntary withdrawal of informed consent. Ultimately, 86 eligible adolescents were enrolled. All participants were screened using strict inclusion and exclusion criteria, and all enrolled adolescents were treatment-naive with no prior exposure to any antidepressants. and randomly allocated into two groups at a 1:1 ratio: the experimental group receiving sequential bilateral rTMS combined with fluoxetine treatment (n=43), and the control group receiving fluoxetine monotherapy combined with sham rTMS intervention (n=43).

During the 4-week intervention period, five participants discontinued the study. In the experimental group, three patients dropped out, including two who withdrew voluntarily owing to recurrent scalp dull pain and one who was lost to follow-up two weeks after intervention initiation. In the control group, two participants withdrew from the study, with one dropping out due to persistent gastrointestinal discomfort and refusal of subsequent blood sample collection, and the other transferring to another medical institution for treatment. The final valid sample for statistical analysis consisted of 81 participants, with 40 cases in the experimental group and 41 cases in the control group.

Baseline demographic, clinical scale, cognitive function and peripheral inflammatory biomarker data are presented in **Table 1**. There were no statistically significant intergroup differences in age, gender composition, years of education, body mass index, disease duration, ASA-C, HAMD-24, WCST indicators, IL-1β, IL-6 and TNF-α (all *P*>0.05), indicating balanced baseline clinical features between the two groups.

**Table 1:** Comparison of baseline characteristics between two groups at baseline.

Baseline characteristics	Experimental group (n=40)	Control group (n=41)	*t*/*χ*²	*P*
Age (years, mean ± SD)	15.87 ± 1.82	15.21 ± 1.64	1.726	0.088
Gender [n (%)]			0.385	0.535
Male	14 (35.00)	17 (41.46)		
Female	26 (65.00)	24 (58.54)		
Education years (years, mean ± SD)	9.62 ± 2.68	9.04 ± 1.93	1.115	0.268
Body mass index (kg/m^2^, mean ± SD)	21.16 ± 2.53	20.42 ± 2.17	1.433	0.155
Disease duration (years, mean ± SD)	3.04 ± 1.03	2.86 ± 1.32	0.685	0.495
ASA-C total score (mean ± SD)	29.15 ± 4.37	28.22 ± 3.84	1.047	0.297
HAMD-24 total score (mean ± SD)	25.13 ± 3.96	24.05 ± 3.51	1.308	0.194
WCST-PCR (%, mean ± SD)	61.48 ± 8.26	63.54 ± 7.19	1.239	0.218
WCST-PCL (%, mean ± SD)	53.72 ± 7.15	55.69 ± 6.63	1.301	0.196
IL-1β (pg/mL, mean ± SD)	14.73 ± 3.41	13.81 ± 2.96	1.305	0.195
IL-6 (pg/mL, mean ± SD)	23.18 ± 4.92	21.95 ± 4.34	1.217	0.226
TNF-α (pg/mL, mean ± SD)	18.75 ± 4.03	17.68 ± 3.61	1.274	0.205

ASA-C, the Chinese Version of the Anhedonia Scale for Adolescents; HAMD-24, the 24-item Hamilton Depression Rating Scale; WCST, Wisconsin Card Sorting Test; PCR, percentage of correct responses; PCL, percentage of conceptualization levels; IL-1β, interleukin-1β; IL-6, interleukin-6; TNF-α, tumor Necrosis Factor-α.

### 3.2 Comparison of ASA-C and HAMD-24 scores before and after treatment

After adjusting for baseline scores via ANCOVA, the experimental group had significantly lower post-intervention ASA-C scores than the control group [*F*_(1, 78)_=35.192, *P* < 0.001], and similarly lower HAMD-24 scores [*F*_(1, 78)_=29.675, *P* < 0.001]. The results are shown in **Table 2**.

**Table 2:** Changes in ASA-C and HAMD-24 scores before and after intervention.

Indicators	Group	Baseline (mean ± SD)	Post-intervention (mean ± SD)	*F*	*P*
ASA-C	Experimental (n=40)	29.15 ± 4.37	20.69 ± 4.01	35.192	<0.001
	Control (n=41)	28.22 ± 3.84	24.95 ± 3.68		
HAMD-24	Experimental (n=40)	25.13 ± 3.96	15.30 ± 4.15	29.675	<0.001
	Control (n=41)	24.05 ± 3.51	19.89 ± 3.74		

ASA-C, the Chinese Version of the Anhedonia Scale for Adolescents; HAMD-24, the 24-item Hamilton Depression Rating Scale.

### 3.3 Comparison of cognitive function and inflammatory cytokine levels before and after treatment

After baseline adjustment via ANCOVA, the experimental group had significantly higher post-treatment WCST-PCR [*F*_(1, 78)_=13.257, *P* < 0.001] and WCST-PCL [*F*_(1, 78)_=10.814, *P* = 0.001] than the control group. Similarly, post-treatment plasma IL-1β [*F*_(1, 78)_=20.146, *P* < 0.001], IL-6 [*F*_(1, 78)_=24.831, *P* < 0.001], and TNF-α [*F*_(1, 78)_=17.528, *P* < 0.001] levels were significantly lower in the experimental group versus the control group after adjusting for baseline values. The results are shown in **Tables 3**, **4**.

**Table 3:** Changes in WCST indicators before and after intervention.

Index	Group	Baseline (mean ± SD)	Post-intervention (mean ± SD)	F	P
PCR (%)	Experimental (n=40)	61.48 ± 8.26	75.19 ± 8.43	13.257	<0.001
	Control (n=41)	63.54 ± 7.19	69.07 ± 7.82		
PCL (%)	Experimental (n=40)	53.72 ± 7.15	67.41 ± 7.68	10.814	0.001
	Control (n=41)	55.69 ± 6.63	61.58 ± 7.03		

WCST, Wisconsin Card Sorting Test; PCR, percentage of correct responses; PCL, percentage of conceptualization levels.

**Table 4:** Changes in inflammatory cytokine levels before and after intervention.

Cytokine	Group	Baseline (mean ± SD)	Post-intervention (mean ± SD)	*F*	*P*
IL-1β(pg/mL)	Experimental (n=40)	14.73 ± 3.41	7.92 ± 2.84	20.146	<0.001
	Control (n=41)	13.81 ± 2.96	11.24 ± 2.76		
IL-6 (pg/mL)	Experimental (n=40)	23.18 ± 4.92	12.15 ± 4.07	24.831	<0.001
	Control (n=41)	21.95 ± 4.34	17.12 ± 4.03		
TNF-α (pg/mL)	Experimental (n=40)	18.75 ± 4.03	10.29 ± 3.36	17.528	<0.001
	Control (n=41)	17.68 ± 3.61	14.35 ± 3.28		

IL-1β, interleukin-1β; IL-6, interleukin-6; TNF-α, tumor Necrosis Factor-α.

### 3.4 Correlation between symptom improvement and inflammatory cytokine reduction

**Figure 1:**
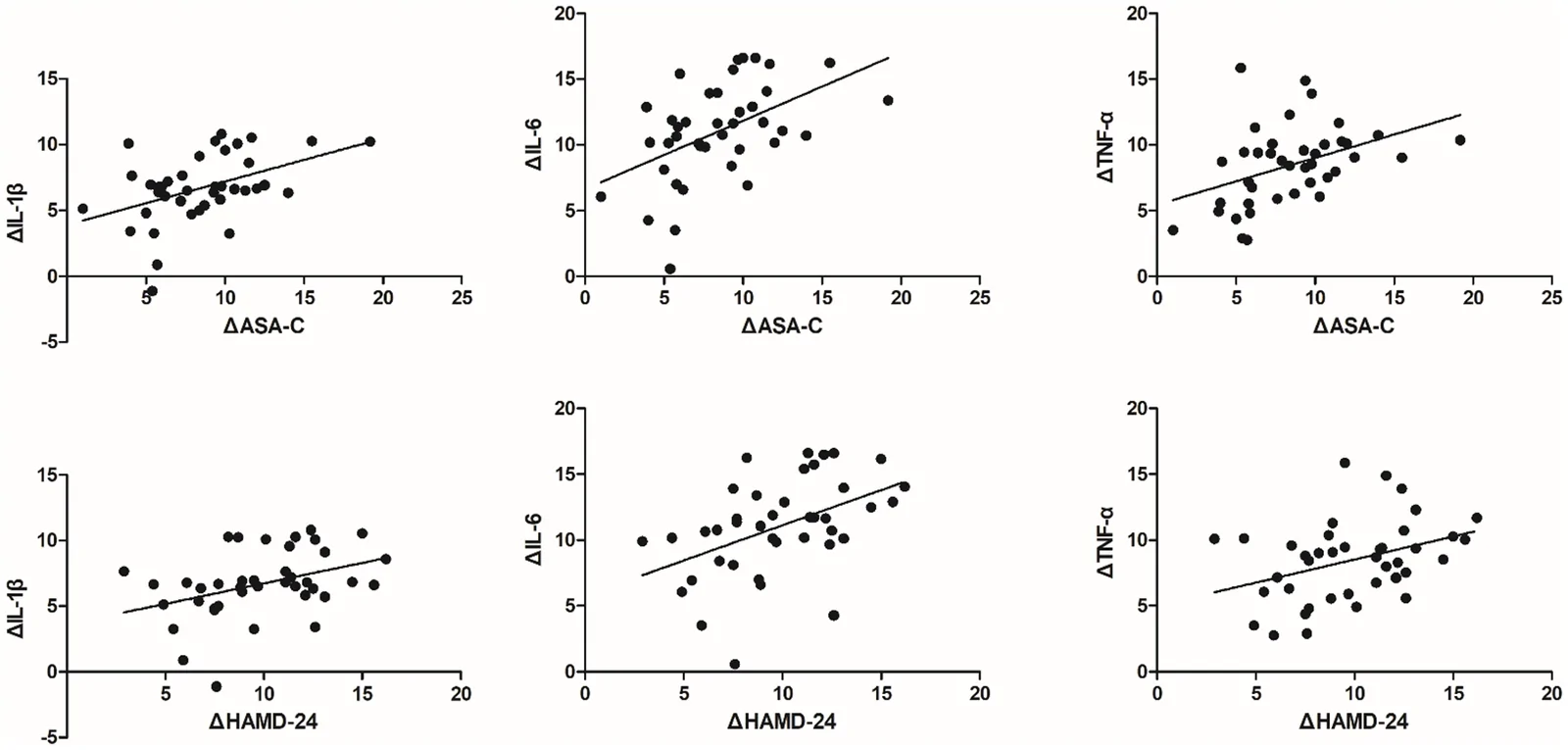
Correlation between symptom improvement and inflammatory cytokine reduction. IL-1β, interleukin-1β; IL-6, interleukin-6; TNF-α, tumor Necrosis factor-α. ASA-C, the Chinese version of the Anhedonia scale for adolescents; HAMD-24, the 24-item Hamilton depression rating scale.

As is shown in **Figure 1**. Pearson correlation analysis was conducted on the change values (Δ = baseline value – post intervention value) of ASA-C, HAMD-24 and three inflammatory cytokines in the experimental group: ΔASA-C was moderately positively correlated with ΔIL-1β (*r* = 0.448, 95%CI: 0.158-0.666, *P* = 0.004), ΔIL-6 (*r* = 0.488, 95%CI: 0.208-0.694, *P* = 0.001), ΔTNF-α (*r* = 0.415, 95%CI: 0.115-0.641, *P* = 0.008); ΔHAMD-24 was weakly to moderately positively correlated with ΔIL-1β (*r* = 0.388, 95%CI: 0.086-0.623, *P* = 0.013), ΔIL-6 (*r* = 0.456, 95%CI: 0.172-0.674, *P* = 0.003), ΔTNF-α (*r* = 0.372, 95%CI: 0.068-0.612, *P* = 0.018).

### 3.5 Adverse events

All adverse reactions recorded during treatment were mild and transient; no severe adverse events including epileptic seizure, persistent severe headache, acute allergic reaction or severe gastrointestinal bleeding occurred in either group. Experimental group (n=40): 9 cases of mild scalp numbness and local headache (22.50%), 3 cases of transient insomnia (7.50%); total adverse event incidence: 30.00%. Control group (n=41): 4 cases of mild nausea and stomach distension (9.76%), 2 cases of mild dizziness (4.88%); total adverse event incidence: 14.63%. Chi-square test revealed no significant difference in overall adverse event rates between two groups (P = 0.079). All mild adverse symptoms in affected participants​ alleviated spontaneously within 1–4 days; no participant required permanent treatment discontinuation.

## 4 Discussion

This 4-week randomized controlled trial demonstrates that adjunctive sequential bilateral rTMS to fluoxetine yields synergistic benefits for adolescents with anhedonic depression, outperforming fluoxetine monotherapy across three key domains: ① greater reduction in core anhedonic symptoms (ASA-C score) and overall depressive severity (HAMD-24 score); ② superior improvement in executive function (WCST indices); and ③ more robust attenuation of systemic proinflammatory cytokines (IL-1β, IL-6, TNF-α). Symptom improvement magnitude correlated moderately with cytokine reductions in the combination group, supporting a shared anti-inflammatory mechanism underlying the enhanced efficacy.

Adolescent depression accompanied by prominent anhedonia poses a critical public health challenge worldwide, and previous evidence indicates that standard SSRI monotherapy often fails to fully resolve reward dysfunction despite partial relief of low mood (6, 26), consistent with the observations derived from the present 4-week randomized controlled trial. This limited therapeutic efficacy of isolated serotonergic intervention aligns with translational frameworks proposed by Pizzagalli (2022), who emphasized that anhedonic pathology extends well beyond central serotonin dysregulation, with chronic peripheral low-grade inflammation and disrupted mesolimbic dopaminergic signaling acting as core mediators of blunted hedonic processing (27). Previous study demonstrated mild anti-inflammatory and neuroplasticity-promoting effects of fluoxetine in rodent depressive models (28), but such biological actions are insufficient to normalize elevated circulating proinflammatory cytokines in clinically affected adolescents (29), which in turn disrupt prefrontal-limbic circuit function and perpetuate anhedonic manifestations (30). Correspondingly, combinatorial regimens incorporating anti-inflammatory agents or anti-inflammatory physical neuromodulation are capable of amplifying the antidepressant and anti-anhedonic therapeutic potency of SSRIs (12).

RTMS has emerged as a well-tolerated non-invasive neuromodulation modality for MDD across age spectrums, yet conventional unilateral high-frequency stimulation targeting the left DLPFC generates inconsistent anhedonia-related outcomes, particularly in patients presenting with inflammatory biomarkers suggestive of immune activation (20). The sequential bilateral rTMS protocol implemented herein first delivers inhibitory 1 Hz stimulation to the hyperactive R-DLPFC, followed by excitatory 10 Hz stimulation over the hypoactive L-DLPFC without interval separation. Prior studies have confirmed that this stimulation paradigm may yield superior antidepressant effects relative to unilateral rTMS protocols (31). Bilateral rTMS produces more therapeutic effects on anhedonia than unilateral stimulation, largely by correcting aberrant interhemispheric excitatory imbalance within the DLPFC. High-frequency rTMS promotes long-term potentiation in the L-DLPFC to facilitate reward and motivational processing, whereas low-frequency rTMS elicits long-term depression in the hyperactive R-DLPFC and attenuates transcallosal inhibitory control over dopaminergic reward circuitry (32). In contrast to unilateral protocols, which merely increase cortical excitability of the L-DLPFC, this bidirectional neuromodulatory strategy strengthens functional connectivity throughout bilateral prefrontal-striatal networks in tandem, recovering physiological neural encoding for both anticipatory and consummatory reward signals (32).

In our study, after adjusting for baseline symptom severity via ANCOVA, adolescents receiving adjuvant bilateral rTMS exhibited significantly lower post-intervention ASA-C and HAMD-24 scores relative to participants receiving fluoxetine monotherapy, confirming robust synergistic antidepressant and anti-anhedonic effects of combined treatment. Cognitive deficits reflected by performance on the WCST represent a prominent functional impairment in adolescents with anhedonic depression, and such deficits are strongly associated with poor academic engagement and social withdrawal (33). Although both treatment groups showed mild improvements in WCST correct response rates and conceptual thinking ability after four weeks of intervention, participants receiving combination treatment exhibited substantially better cognitive recovery outcomes. rTMS rescues cognitive deficits by elevating synaptic plasticity, restoring glutamatergic-GABAergic excitatory-inhibitory balance, and normalizing dysfunctional frontoparietal and default mode network synchronization to facilitate memory encoding and executive control (34).

Recent translational studies utilizing inflammatory challenge paradigms have identified circulating IL-1β, IL-6, and TNF-α as well-validated peripheral biomarkers that link systemic inflammation to hedonic deficits among adolescents with depression (35). At the four-week timepoint, significant within-subject declines in all three proinflammatory cytokines were detected under both interventions. Suppression of these inflammatory factors was substantially more pronounced for individuals who received add-on sequential bilateral rTMS.

Correlation analysis restricted to the combination treatment group revealed moderate positive associations between the magnitude of symptomatic improvement (ΔASA-C, ΔHAMD-24) and the magnitude of reduction in each proinflammatory cytokine, indicating an association between reduced peripheral inflammation and alleviated depressive and anhedonic symptoms. Classical SSRIs only elicit modest anti-inflammatory effects via regulating inflammatory transcription factors, but co-administration with bilateral rTMS strongly boosts such bioactivity. This neuromodulatory approach suppresses overactive brain microglia and lowers peripheral cytokine levels at the same time. Their combined action creates a synergistic anti-inflammatory response to reverse inflammation-related dysfunction within the reward circuitry (19, 36, 37). These biomarker-derived findings provide a mechanistic explanation for the superior clinical efficacy of combined intervention, and support the application of peripheral IL-1β, IL-6 and TNF-α as objective laboratory indicators to monitor therapeutic response in adolescent anhedonic depression clinical practice.

All adverse events captured throughout the trial period were mild, transient and self-resolved within one to four days without permanent treatment discontinuation, and no severe adverse outcomes. The experimental group displayed a numerically higher overall adverse event incidence (30.00%) relative to the control group (14.63%), though intergroup disparity failed to reach statistical significance. Stimulation-specific adverse reactions observed in the rTMS combination cohort primarily consisted of mild scalp paraesthesia, localized cephalalgia and transient insomnia, consistent with the well-established safety profile of standard rTMS paradigms summarized in previous clinical studies (38).

This study has several notable limitations. First, the sham protocol employing a 90°coil configuration poses a substantial risk of unblinding. Active stimulation necessitates tight contact between the coil and scalp, which generates palpable vibratory sensations; by contrast, sham coils are positioned without such close scalp apposition. Clinically relevant sensory discrepancies were confirmed by participant self-reporting: 22.5% of individuals allocated to the active stimulation group reported scalp paraesthesia, whereas no participants in the sham group noted comparable sensory symptoms. We implemented multiple strategies to minimise assessment bias, including strict blinding of all outcome evaluators, standardised pre-study briefing to inform all enrolled participants that scalp discomfort could potentially arise in either trial arm, and independent blinded rating of all outcome metrics. Even with these safeguards in place, residual bias affecting subjectively reported ASA-C scale scores cannot be ruled out. For this reason, we interpret raw within-group changes derived from participant self-report data with considerable circumspection, and prioritise adjusted intergroup mean differences as more robust indicators of treatment efficacy. This methodological limitation warrants resolution in subsequent paediatric clinical trials, which should adopt active sham coils engineered to deliver sensory feedback matched to that produced by real stimulation coils. Second, we performed six pairwise correlation analyses linking three inflammatory cytokines (IL-1β, IL-6, TNF-α) to two clinical rating scales (ASA-C, HAMD-24) without applying any formal multiple comparison correction. Though these correlational tests were framed as exploratory analyses meant to generate mechanistic hypotheses, the risk of inflated Type I error (false positive results) remains present. Accordingly, all observed correlation coefficients should be regarded as preliminary evidence only. Larger, dedicated confirmatory studies incorporating suitable multiplicity correction methods such as the False Discovery Rate are needed to validate these associations. Third, the absence of a double-sham control arm limits disentangling rTMS-specific effects from time, natural remission, or non-specific effects.​ The control group’s significant ASA-C reduction (28.22 → 24.95) likely reflects fluoxetine efficacy, spontaneous symptom fluctuation, and trial-related psychosocial benefits. Thus, while between-group differences support rTMS’s additive value, absolute score changes require cautious interpretation. Fourth, the asymmetric pulse dosing (3,000 pulses for L-DLPFC vs. 900 pulses for R-DLPFC) introduces a potential confounder for mechanistic interpretation, as it remains unclear whether clinical benefits derive primarily from left excitatory stimulation, right inhibitory effects, or their interactive network-level modulation. Future dose-finding studies are warranted to isolate hemisphere-specific contributions. Fifth, we only adopted a four-week acute treatment schedule and did not arrange long-term follow-up after intervention. As such, we could not track sustained symptom relief, long-term cognitive preservation, or dynamic shifts in peripheral inflammatory markers once treatment was stopped. Sixth, our detection panel only covered three common peripheral pro-inflammatory cytokines. Future multi-omics work that incorporates chemokines, neurotrophic factors and cerebrospinal fluid biomarkers will help fully unpack how sequential bilateral rTMS modulates central neuroinflammatory pathways.

In conclusion, adjunct sequential bilateral rTMS yields synergistic benefits for adolescent anhedonic depression by ameliorating core symptoms, restoring executive function and attenuating systemic inflammation. Peripheral IL-1β, IL-6 and TNF-α are viable biomarkers for therapeutic monitoring, and this combined regimen is safe for adolescents with residual anhedonia after fluoxetine monotherapy. Notably, while these 4-week findings demonstrate robust acute efficacy, longitudinal follow-up is warranted to evaluate relapse rates and the durability of treatment effects in this vulnerable population.​ Future large-scale studies incorporating extended observation periods and double-sham control arms are needed to confirm these preliminary findings and guide long-term clinical implementation.

## Data Availability

The raw data supporting the conclusions of this article will be made available by the authors, without undue reservation.
